# New Horizons in Neuroscience: The Summer School of Brain Mapping and Stimulation Techniques

**DOI:** 10.25122/jml-2025-1001

**Published:** 2025-04

**Authors:** Livia Livinț-Popa, Hanna Dragoș, Irina Vlad, Victor Dăbală, Vlad Chelaru, Emanuel Ștefănescu, Bianca Crecan-Suciu, Dafin Mureșanu

**Affiliations:** 1 Department of Neurosciences, Iuliu Hațieganu University of Medicine and Pharmacy, Cluj-Napoca, Romania; 2 RoNeuro Institute for Neurological Research and Diagnostic, Cluj-Napoca, Romania; 3 County Emergency Clinical Hospital, Cluj-Napoca, Romania; 4 Faculty of Medicine, Iuliu Hațieganu University of Medicine and Pharmacy, Cluj-Napoca, Romania

## INTRODUCTION

Brain mapping has evolved as an experimental discipline that combines concepts from neuroscience, neuroimaging, psychology, psychiatry, technology, engineering, and artificial intelligence [1-7]. By transforming biological signals into clear data, neurotechnologies have advanced the understanding of the human brain, offering unprecedented insights into its anatomy and function, ultimately improving medical practice [8-11]. As research increasingly focuses on functional brain organization, specialists in neurology and related fields benefit from a better understanding of these fundamental concepts.

Neurotechnology is essential to for identifying and understanding neural organization and functioning, related to the ability to observe brain function in real-time can aid in diagnosing and treating various neurological diseases [11-12], and lay the groundwork for innovative applications, including brain mapping, thus revolutionizing our understanding of the brain and brain health [10-13]. Below are some of the most proeminent brain mapping technology for neuroscience research [13-15].

With notable advancements over the years, electroencephalography (EEG) and quantitative electroencephalography (QEEG) both analyze electrical patterns in the brain, providing information on its functions. EEG works by registering brain waves and evaluating their patterns, while QEEG uses complex algorithms to transform them into numerical data, which can improve understanding of neural networks and functional connectivity. Although these techniques are most commonly used for evaluating epilepsy, they can also be applied to stroke, traumatic brain injury, and neuropsychiatric disorders [12-14,16].

Furthermore, eye tracking (ET) is an emerging technology that offers biological, cognitive, and behavioral insights into brain processes by quantifying and analyzing eye movements. Over the years, it has been increasingly used in medical research and beyond, with applications in psychology, rehabilitation, and other non-medical domains. By evaluating gaze patterns, saccades, and fixations, ET offers valuable information on perception and information processing [11].

Notably, non-invasive brain stimulation techniques, such as repetitive transcranial magnetic stimulation (rTMS) and transcranial direct current stimulation (tDCS), are often used as supportive therapies in treating neurological and psychiatric disorders, particularly in patients who do not respond well to conventional treatments. In recent decades, research has shown their expanding applications in treating various disorders, such as major depressive disorder, chronic pain, and stroke, as well as for cognitive enhancement [17-19].

Finally, functional magnetic resonance imaging (fMRI) [6,9] and functional near infra-red spectroscopy (fNIRS) [6,8] are pivotal non-invasive technologies for brain mapping, assisting researchers to identify active brain regions during specific tasks by detecting changes in blood oxygenation levels. Magnetoencephalography (MEG) measures small magnetic fields generated by neuronal activity and is ideal for studying rapid neurological processes [20], which require timely resolution.

As society shifts from a single-disciplinary approach to a multidisciplinary model, neuroscience showcases the importance of converging perspectives. Understanding the complexities of the most advanced network – the human brain – is not a one-dimensional process; it requires expertise on multiple levels [21]. Whether applied in education, research, or clinical practice, this model can lead to innovation and benefit the world at large [21-22].

## SUMMER SCHOOLS IN CLUJ-NAPOCA: TOWARDS A LEGACY OF NEUROTECHNOLOGICAL EDUCATION

The Summer Schools in Cluj-Napoca, focused on the use of neurotechnology in brain mapping and neurostimulation, are gaining value for neurotechnology education, in line with with the broader mission of enhancing international, multidisciplinary scientific collaboration and learning.

Coordinated by Prof. Dr. Dafin Mureșanu and Lecturer Dr. Livia Popa, the events were conducted under the umbrella of Neurotech^EU^, the European University of Brain and Technology, and the Erasmus+ Programme. Hosted at two prestigious Romanian institutions – the University of Medicine and Pharmacy Iuliu Hatieganu (UMFIH) and RoNeuro Institute for Neurological Research and Diagnostic – the events integrated online lectures, on-site presentations and hands-on sessions [23, 24].

Neurotech^EU^, the European University of Brain and Technology, represents an alliance of eight European universities that strives to create a complex European network that fosters progress in neuroscience and technology through academic exchanges and collaboration. Neurotech^EU^ aims to ensure the integration of stakeholders’ perspectives from scientific and research fields, but also governmental and non-governmental organizations, and patient advocacy groups, thus promoting multidimensional approaches to neuroscience [25].

The University of Medicine and Pharmacy Iuliu Hatieganu (UMFIH) from Cluj-Napoca is a prestigious Romanian institution of higher education, with a tradition spanning more than 150 years in the medical field. UMFIH is a modern entity that supports multiculturalism, excellence in academia, and advanced research, striving to cultivate young specialists equipped to excel in the medical sector. The university is renowned not only for its excellence in teaching but also for its contributions to translational and clinical research, carried out in hospitals and specialized centers, including:

- Centre for Translational Research in Neurosciences;
- Centre for Research on the Dissemination of Drug-Related Information;
- Centre of Research in Functional Genomics, Biomedicine and Translational Medicine;
- Centre for Experimental Medicine and Practical Skills;
- MedFUTURE – the Research Centre for Advanced Medicine [26].

RoNeuro Institute for Neurological Research and Diagnostic closely collaborates with UMFIH and Neurotech^EU^ and is committed to advancing education, patient care, and neurological research – values closely aligned with those of UMFIH and NT^EU^. RoNeuro Institute actively engages in patient care and scientific research on prevalent neurological diseases (e.g., cerebrovascular diseases, neurotraumatology, cognitive disorders, neurorehabilitation, transcranial sonography, and neuroepidemiology), while fostering research groups on EEG, QEEG, TMS, and ET. By integrating clinical practice with pioneering research, RoNeuro Institute fulfils its core mission of enhancing patient care and contributing to the advancement of neurology [27].

The first two editions laid the foundation for what is now a growing legacy in the field [23-24].

"As co-organizer of both the Summer School of Brain Mapping and Stimulation Techniques and the previous editions of the Summer School of Quantitative Electroencephalography (QEEG), I am truly proud of how these programs have evolved. Each edition has strengthened our commitment to fostering hands-on learning and interdisciplinary collaboration in neurotechnology. Seeing students and young researchers from diverse backgrounds engage with cutting-edge brain mapping techniques, exchange knowledge, and build professional networks is incredibly rewarding. The enthusiasm and curiosity they bring to the program reaffirm the importance of such initiatives in shaping the next generation of neuroscientists."

*Lecturer Dr. Livia Popa*

### First Edition (2022)

The inaugural edition of the European Summer School of Quantitative Electroencephalography (QEEG) was held in Cluj-Napoca from July 11^th^ to 15^th^, 2022. This event marked the first Blended Intensive Program (BIP) within the Neurotech^EU^ alliance, co-organized by the UMFIH and RoNeuro Institute for Neurological Research and Diagnostic. The primary aim of this event was to introduce junior researchers from across Europe to QEEG, providing them with theoretical knowledge and hands-on experience. Over five days, participants from six European countries were immersed in courses on the basics of EEG, moving on th advanced QEEG analysis, including the use of QEEG in conjunction with other neurotechnologies promoting a multidisciplinary exchange of skills and ideas [23].

### Second Edition (2023)

Following the success of the first edition, the second QEEG Summer School took place from July 17^th^ to 21^st^, 2023, also in Cluj-Napoca. Building upon the curriculum of the first edition, this program covered both fundamental and advanced topics such as brain connectivity analysis, TMS and ET, as well as the potential integration of QEEG with other neurotechnologies. The summer school continued to emphasize hands-on, collaborative learning, with participants working on multidisciplinary group projects where they applied the knowledge acquired throughout the program. The event attracted participants from a wider range of institutions, reinforcing the collaborative spirit of Neurotech^EU^ and expanding the reach of neurotechnological education [24].

### The 2024 Edition: Continuity and Evolution

The 2024 event aimed to further build on the foundations set by the first two editions, with an enhanced curriculum focusing on hands-on experience on neurotechnologies and integrating theory with practical applications. Participants explored other cutting-edge brain mapping technologies, such as fMRI, fNIRS, and MEG, from a theoretical perspective. The program was designed to equip partakers with both basic knowledge and practical skills needed for brain mapping, while fostering collaboration and innovation across disciplines in neuroscience, considering the varied backgrounds of the participants.

“The Erasmus+ program on brain mapping and stimulation in Cluj-Napoca, Romania, was an extraordinary experience. The hands-on training with advanced tools like fMRI, EEG, and TMS deepened my knowledge and skills in neuroscience. The instructors were experts, providing clear guidance on both theory and practical applications. Working alongside international peers in Cluj’s vibrant academic environment fostered invaluable connections and collaboration. The city itself, rich in history and culture, offered a warm and inspiring backdrop. I highly recommend this program to anyone seeking to expand their expertise in brain research while enjoying an unforgettable cultural experience.”

*Stavroula Besina, participant*

## THE SUMMER SCHOOL OF BRAIN MAPPING AND STIMULATION TECHNIQUES: TRAINING A NEW GENERATION OF NEUROSCIENTISTS

The Summer School of Brain Mapping and Stimulation Techniques, developed through Erasmus+ and Neurotech^EU^, bought together 28 students from eight European universities in a blended learning format. This included a mix of online sessions (held from 3-4 September) and on-site sessions hosted by UMFIH and RoNeuro Institute for Neurological Research and Diagnostic (9-13 September), offering an introduction to neurotechnologies.

Coordinated by Prof. Dr. Dafin Mureșanu and Lecturer Dr. Livia Popa, the educational event welcomed participants from Bonn University (Germany), Lille University (France), Karolinska Institute (Sweden) Medical University of Sofia (Bulgaria), Ankara Yildirim Beyazit University (Turkey) , Radboud University (the Netherlands) , Eötvös Loránd University (Hungary), and the University of Medicine and Pharmacy Iuliu Hatieganu from Cluj-Napoca (Romania). Medical students, residents, and Master's and Ph.D. students, specializing in medicine, neuroscience, medical technology, healthcare business, bioentrepreneurship, cognitive neuroscience, and psychology were immersed in the field of brain mapping and stimulation techniques over the seven days.

**Figure 1:**
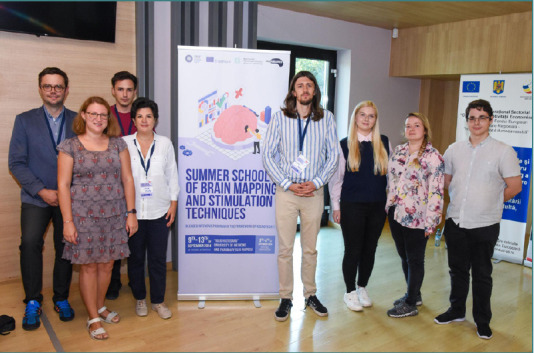
The representatives of the local scientific team of the Summer School of Brain Mapping and Stimulation Techniques

The program was supported by the local scientific team – Prof. Dr. Dafin Fior Mureșanu, Lecturer Dr. Livia Popa, Dr. Hanna Dragoș, Dr. Victor Dăbală, Dr. Vlad Chelaru, Dr. Irina Vlad, Dr. Emanuel Ștefănescu, Dr. Olivia Verișezan-Roșu, Dr. Bianca Crecan-Suciu, Dr. Georgiana Novăceanu, Lecturer Alexandrina Tomoioagă, and Lecturer Horațiu Crișan (Figure 1). Top-tier technical equipment used during the sessions included:

- a complete equipment for EEG recording on 21-channels (Neurosoft – LIAMED), respectively 32 channels (NicoletOne™ EEG system – Natus Medical);
- Magpro X100 stimulator from Magventure Denmark for the TMS session;
- PlatoWork tDCS headset;
- Tobii ProTX300 remote eye tracker.

Furthermore, all data was analyzed in Brainstorm, BrainVision Analyzer 2.2, and R Statistical Software.

Lastly, he offered some considerations for overcoming limitations in neurorehabilitation.

### Breakdown of Activities

Prof. Dr. Dafin Mureșanu opened the event with a warm welcome to the participants and a presentation on the paradigms of brain technology in neuroscience. In his presentation, he emphasized the importance of constructing a multilevel, integrative approach to neurosciences – one that is based on collaboration among scientists and a diverse range of stakeholders including governments, companies, patient advocacy organizations, health systems, to private research institutions, scientific societies, etc. Prof. Mureșanu introduced the participants to Neurotech^EU^ and its partnering institutions, UMFIH and RoNeuro Institute, highlighting their shared aim to promote and sustain education, research, and international collaboration. As the field of brain mapping and stimulation still faces a knowledge gap, the objective of the Summer School was to initiate and expand the grasp on the subject by educating participants on the underlying mechanisms, actual and potential uses, as well as future opportunities.

**Figure 2:**
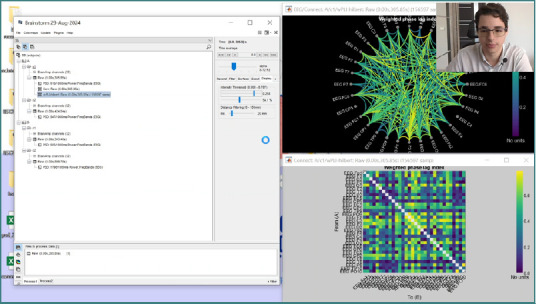
Presentation in Brainstorm by Dr. Vlad Chelaru

**Figure 3:**
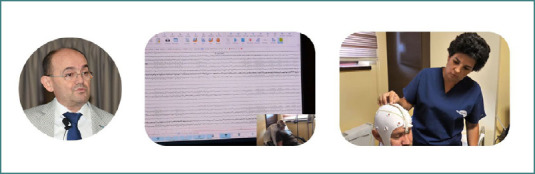
Professor Dafin Muresanu; Lecturer Dr. Livia Popa demonstrating an EEG procedure during the online sessions from the Summer School of Brain Mapping and Stimulation Techniques

On September 3^rd^-4^th^, 2024, participants attended online sessions structured around the basics of EEG, QEEG, and advanced neuroimaging techniques, including fMRI, fNIRS, MEG. Additionally, they were introduced to ET and non-invasive brain stimulation techniques, such as TMS and tDCS. The presentations were coordinated by Lecturer Dr. Livia Popa, Dr. Hanna Dragoș, Dr. Victor Dăbală, Dr. Vlad Chelaru (Figure 2), and Dr. Irina Vlad. The sessions began with an introduction to the neurobiology of EEG signals and the steps involved in performing an EEG, accompanied by a live EEG registration (Figure 3). Following this, the lecturers covered the basics of QEEG analysis and its clinical applications, walking participants through the QEEG Analysis Pipeline (From NicVue to BrainVision Analyzer & Brainstorm). Students were also introduced to Brainstorm and data analysis using R software. The final part of the virtual program focused on the integration of QEEG with other neurotechnologies, such as fMRI, fNIRS, TMS, tDCS, transcranial alternating current stimulation (tACS), MEG, and ET.

The students were encouraged to ask questions throughout the sessions, and the lecturers provided additional clarifications and clinical examples to help them better comprehend the concepts and grasp the intricacies of the field from seasoned professionals. Each day concluded with a review session to consolidate the information covered.

More information about the Summer School of Brain Mapping and Stimulation Techniques will be presented in a further editorial.
